# Absence of PD-L1 on tumor cells is associated with reduced MHC I expression and PD-L1 expression increases in recurrent serous ovarian cancer

**DOI:** 10.1038/srep42929

**Published:** 2017-03-07

**Authors:** Stefanie Aust, Sophie Felix, Katharina Auer, Anna Bachmayr-Heyda, Lukas Kenner, Sabine Dekan, Samuel M. Meier, Christopher Gerner, Christoph Grimm, Dietmar Pils

**Affiliations:** 1 Dept. of Obstetrics and Gynecology, Comprehensive Cancer Center (CCC), Medical University of Vienna, Vienna, Austria; 2 Dept. of Pathology, Medical University of Vienna, Vienna, Austria; 3 Dept. of Analytical Chemistry, University of Vienna, Vienna, Austria; 4 Section for Clinical Biometrics, Center for Medical Statistics, Informatics, and Intelligent Systems (CeMSIIS), Medical University of Vienna, Vienna, Austria; 5 Dept. of Surgery, Medical University of Vienna, Vienna, Austria

## Abstract

Immune-evasion and immune checkpoints are promising new therapeutic targets for several cancer entities. In ovarian cancer, the clinical role of programmed cell death receptor ligand 1 (PD-L1) expression as mechanism to escape immune recognition has not been clarified yet. We analyzed PD-L1 expression of primary ovarian and peritoneal tumor tissues together with several other parameters (whole transcriptomes of isolated tumor cells, local and systemic immune cells, systemic cytokines and metabolites) and compared PD-L1 expression between primary tumor and tumor recurrences. All expressed major histocompatibility complex (MHC) I genes were negatively correlated to PD-L1 abundances on tumor tissues, indicating two mutually exclusive immune-evasion mechanisms in ovarian cancer: either down-regulation of T-cell mediated immunity by PD-L1 expression or silencing of self-antigen presentation by down-regulation of the MHC I complex. In our cohort and in most of published evidences in ovarian cancer, low PD-L1 expression is associated with unfavorable outcome. Differences in immune cell populations, cytokines, and metabolites strengthen this picture and suggest the existence of concurrent pathways for progression of this disease. Furthermore, recurrences showed significantly increased PD-L1 expression compared to the primary tumors, supporting trials of checkpoint inhibition in the recurrent setting.

Epithelial ovarian cancer (EOC) is a complex and therapeutically challenging malignancy, diagnosed with advanced disease and extensive peritoneal tumor spread in more than 70% of patients. Despite initial response to standard therapy (advanced surgery followed by platinum based chemotherapy) clinicians as well as patients have to face drug-resistance, early tumor-recurrence, extensive malignant ascites and a high mortality.

New therapeutic approaches are urgently needed for advanced and recurrent EOC, especially for the two different types of high grade serous ovarian cancer, presenting with different modes of peritoneal tumor spread, miliary or non-miliary^1^,^2^,^3^,^4^, which arise potentially from different origins, fallopian tubes or ovarian inclusion cysts^1^,^5^. The importance of the tumor immune microenvironment in EOC^6^,^7^ has been demonstrated in various trials evoking high expectations for tumor specific immunotherapy^8^,^9^. Cytotoxic T lymphocytes - key players in antitumor activity - are actively inhibited by immune checkpoint molecules. Up-regulation of programmed death-1 (PD-1) and its ligand PD-L1 mediates immune suppression by inactivating T lymphocytes together with multiple concurrent mechanisms^10^. Expression of PD-L1 is regulated by oncogenes and induced by various proinflammatory molecules in the tumor microenvironment^11^. Currently, promising activity of antibodies targeting PD-1 and PD-L1 across multiple malignancies^12^ raise expectations on the role of these agents also in EOC. But so far, clinical data on the effects of immune checkpoint inhibitors tested as single agents in relapsed EOC patients shows an overall response rate of maximum 15%^13^,^14^,^15^.

Combination with chemotherapy is under investigation in various trials but sufficient biological data is still missing to define patients amendable to immune checkpoint inhibitors. In order to evaluate if primary or recurrence would be equally eligible for PD-L1 targeted therapy, expression values were compared in tumor tissue acquired at the time of primary diagnosis *versus* tumor recurrence. Trying to understand biological and immunological processes associated with PD-L1 expression in serous EOC, whole sections from the ovarian tumor and from peritoneal implants were used to determine PD-L1 expression patterns and analyzed together with comprehensive cyto- and chemokine data, metabolomics, RNA sequencing data, and flow cytometric data.

## Results

### Expression patterns of PD-L1 in HGSOC tumor tissues – impact on survival

**Figure 1:**
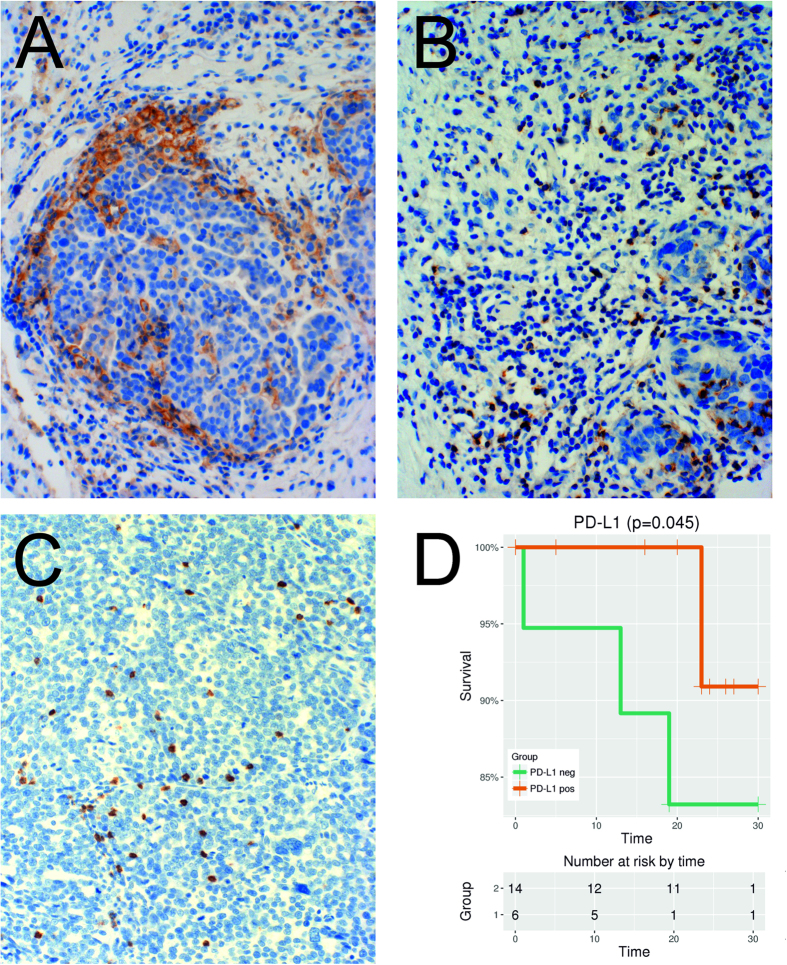
(**A**) Immunohistochemistry (IHC) of PDL1, 100×: Tumor cells stain positively for PDL1 mainly at the tumor-stroma border. (**B**) IHC of PD1, 100×: At the tumor-stroma border clusters of immune cells and TILs with PD1 positive cytoplasmatic +/− membrane staining were observed. (**C**) IHC of CD8, 100×: CD8 positive TILs infiltrating the tumor. (**D**) The positive impact of PD-L1 expression (≥1% *versus* 0%) on overall survival (months) shown by a Kaplan-Meier estimate (y-axis is truncated to see the difference more pronounced; p = 0.045; Log-rank test).

Complementing the data obtained from TMAs by Webb *et al*.^16^, analysis of whole tissue sections (WTSs) revealed that only very few EOC tumor cells express PD-L1. 24 WTSs from solid primary ovarian tumors (P) and 20 WTSs from metastatic implants (M) within the peritoneal cavity, mainly from tumors within the omentum majus, obtained from 28 patients were analyzed (Table 1). Both, P and M tumor tissues had the same focal staining pattern, whereby mainly tumor cells at the tumor-stroma border stained positively for PD-L1 (Fig. 1A). PD-L1 expression was almost exclusively observed on tumor cells with adjacent tumor infiltrating lymphocytes (TILs). A membranous +/− cytoplasmic staining was observed. Tumors were scored as PD-L1 positive if ≥1% of tumor cells within the whole tumor area stained positive for PD-L1.

Comparing the whole tumor areas within the WTSs of P and M revealed that 75% (n = 18 with the following percentage of positively stained tumor cells: 1%, n = 3; 5%, n = 13; 10%, n = 2) of the samples from the ovarian tumor were PD-L1 positive, compared to 40% (n = 8: 1%, n = 1; 5%, n = 6; 20%, n = 1) of the samples from metastatic implants (p = 0.019). Out of the 18 PD-L1 positive samples (P), the maximum of PD-L1 positive tumor cells was 10% (n = 2). Within the metastatic implants a maximum of 20% positive tumor cells was seen in one sample - the matched P tissue (Pat-ID 53, Table 1) had 10% PD-L1 positive tumor cells.

In Fig. 1D a Kaplan-Meier estimate is shown indicating a positive impact of the PD-L1 abundance (≥1% versus 0%) on overall survival (p = 0.045; Log-rank test), albeit supported by only few samples but in accordance to most evidences in ovarian cancer^16^,^17^ which is in discordance to the impact of PD-L1 expression on survival in nearly all other cancer entities including gastric cancer, hepatocellular carcinoma, renal cell carcinoma, esophageal cancer, pancreatic cancer, and bladder cancer^18^. The significant positive impact of PD-L1 expression holds also in a multiple Cox-regression analysis correcting individually for age (p = 0.049) and as a trend when correcting for residual tumor (p = 0.079).

### TIL frequency correlates with PD-L1 expression

A significantly higher frequency of CD8+ TILs was seen in WTSs with PD-L1 expressing tumor cells, both in primary (p = 0.011; n = 24) and metastatic tumor tissues (p = 0.037; n = 20) (Suppl. Figure S1). Clusters of immune cells were frequently observed at the tumor-stroma border; clustering was also – but less frequently – seen directly within the tumor tissue. The only sample without CD8+ cell infiltration was a metastatic implant also negative for PD-L1 expressing tumor cells (Pat ID 85).

5 out of 44 WTSs were negative for PD-1 (one P and four M). All of them were also negative for PD-L1 expressing tumor cells. Among them was the one sample (M, Pat ID 85) also negative for CD8.

### Expression analysis reveals three deregulated pathways in immune signaling

**Figure 2:**
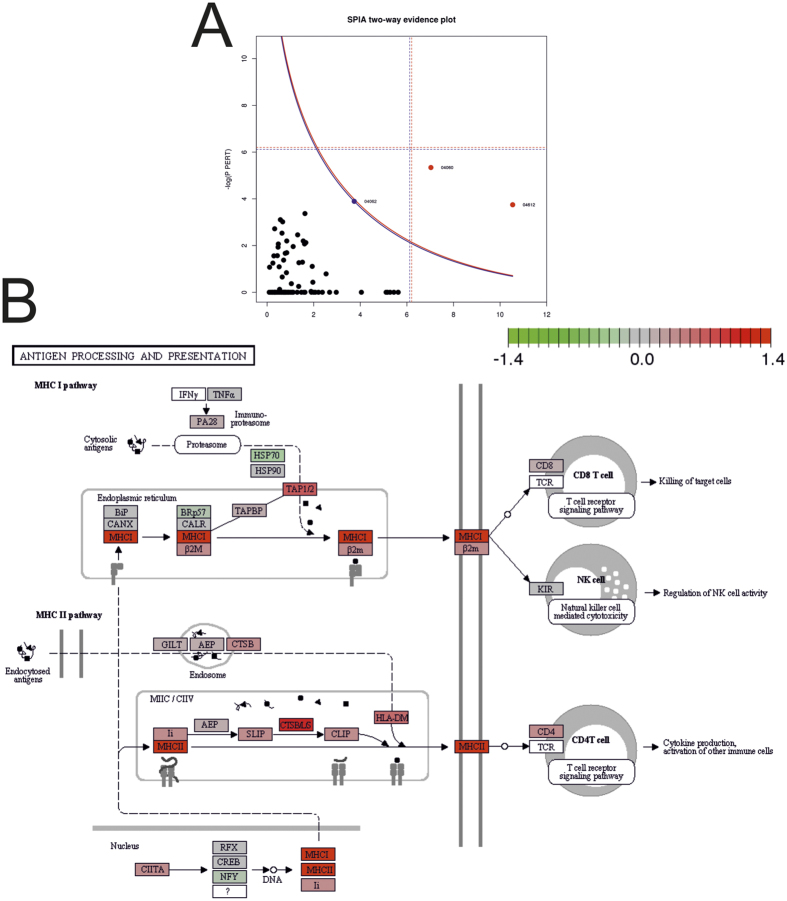
(**A**) Signaling Pathway Impact Analysis (SPIA) evidence plot (blue curve, FDR 10% cut-off; *cf.* Table 2) and (**B**) the KEGG pathway-plot for the “Antigen processing and presentation” pathway (ID 4612; color of nodes: red, positively correlated and green, negatively correlated to the PD-L1 abundance). (http://www.kegg.jp/kegg/kegg1.html^ ^19).

974 genes (thereof 708, 72.6%, protein coding) in isolated tumor cells were significantly (FDR 20%) correlated to the PD-L1 abundance, thereof 488 negatively and 486 positively. A KEGG pathway^19^ analysis of these differentially expressed genes using SPIA yielded three deregulated pathways (Table 2, Fig. 2A) namely (i) chemokine signaling pathway, (ii) cytokine-cytokine receptor interaction (Suppl. Fig. S2), and (iii) antigen processing and presentation (MHC I class, MHC II class) (Fig. 2B). The chemokine signaling pathway and the cytokine-cytokine receptor interaction pathway indicate a local immunological activation.

**Figure 3:**
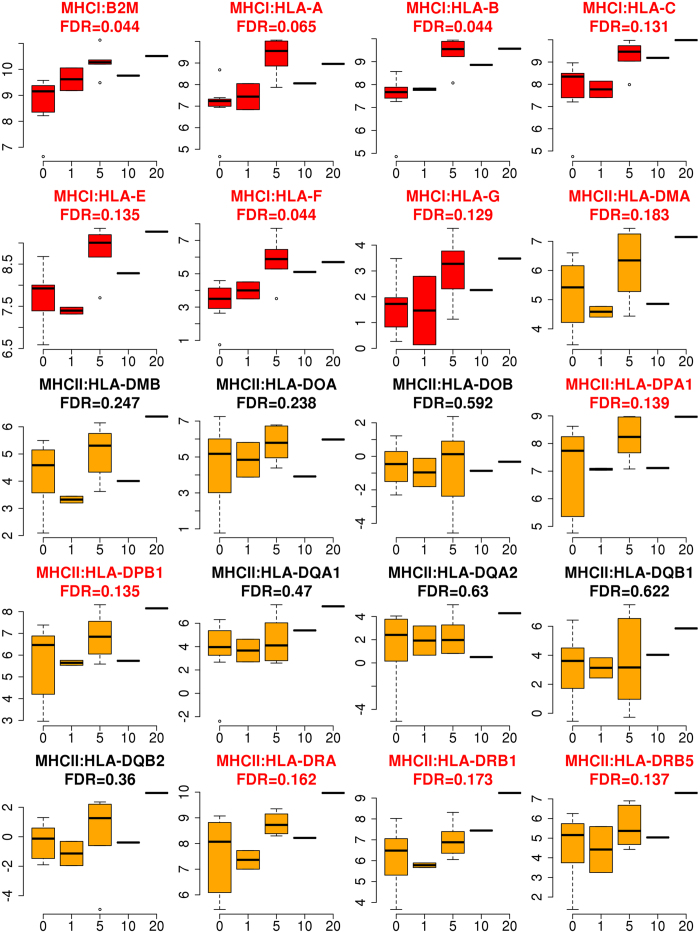
Association between PD-L1 expression (x-axis) and HLA-coding genes (y-axis). Red boxplots indicate MHC I class protein-coding genes and orange boxplots MHC II class protein-coding genes. Red headings indicate significance (FDR < 20%).

In-detail analysis of the KEGG pathway MHC I + II class genes (112 HLA genes) showed that out of 19 reliably expressed coding genes, six differentially expressed genes were from the MHC I class path - all of them significantly positively correlated, and 13 were from the MHC II class path (six significantly positively correlated, 46%; Fig. 3). Out of eleven reliably expressed non-coding genes only one was significant (9%) (Suppl. Fig. S3). Significantly positively correlated genes of the MHC-I class path were HLA-A, HLA-B, HLA-C, HLA-E, HLA-F, HLA-G, and HLA-DMA and of the MHC-II class path HLA-DPA1, HLA-DPB1, HLA-DRA, HLA-DRB1, and HLA-DRB5 (Fig. 3). Additionally, the B2M gene – a component of the MHC-I class protein complexes, coded unlike the HLA protein genes on chromosome 15 – encoding for the β2-microglobulin protein, was also significantly positively correlated to PD-L1 expression (Fig. 3). Summarized, the expression of all expressed MHC I genes, including B2M, are significantly positively correlated to the PD-L1 abundance in tumor cells.

### Association between PD-L1 and systemic inflammatory processes

To determine the relationship between systemic inflammatory processes and tumor cell PD-L1 abundance, a quantitative analysis of serum cyto- and chemokines was performed. The first set of Luminex-panels revealed seven analytes positively (sHER2neu, CCL22, Leptin, sEGFR, GM-CSF, PECAM1, and MIF) and two analytes negatively (CCL23 and CXCL11) associated with PD-L1 expression (accepting an FDR of 20%) and the second set revealed three negatively associated cytokines (IL5, IL2R, and IL10) (*cf.* Materials and Methods, Table 3 and Suppl. Fig. S4).

### PD-L1 associated with immune cell populations determined by flow cytometry

**Figure 4:**
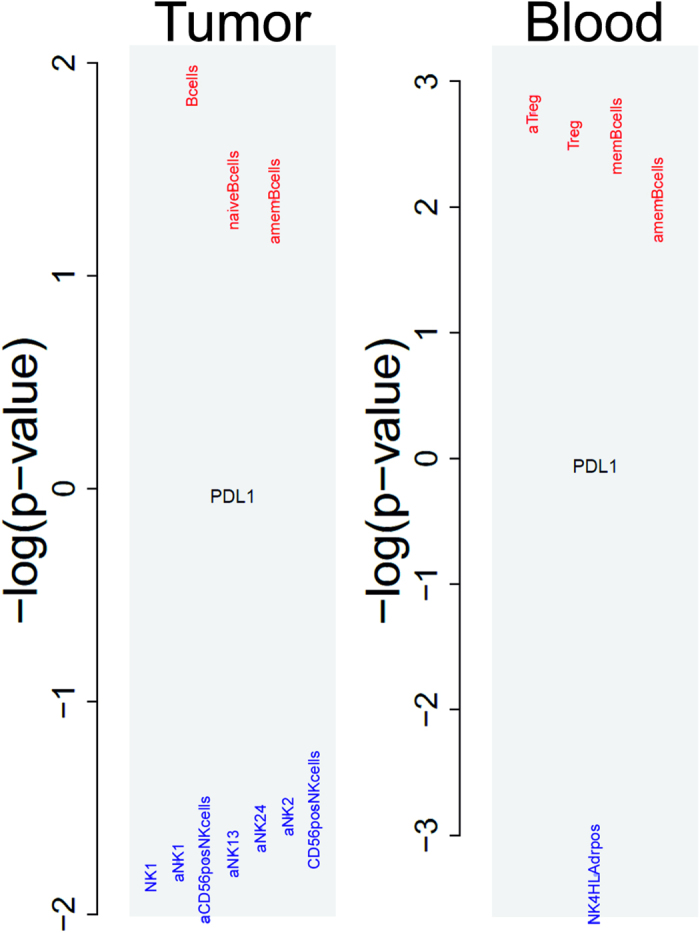
Association of PD-L1 tumor cell abundances and immune cell populations in tumor tissues and blood determined by flow cytometry. In tumor tissues B-cells (comprising naïve and memory B-cells) showed a positive and CD56 positive NK cells including different sub-populations (NK1 and NK2) a negative association. In blood, regulatory T-cells and memory B-cells showed a positive and HLA-DR positive NK4 cells a negative correlation. (“a.” before the cell population name means absolute values, otherwise relative frequencies; y-axis, negative log_10_ p-values – if positive, a positive correlation, if negative, a negative correlation is indicated.

Correlating PD-L1 abundance with flow-cytometric data obtained from EpCAM depleted EOC tumor tissue cells allows a better understanding of associated local immunological players. As shown in Fig. 4, PD-L1 abundance was positively associated with tumor associated B-cells (comprising naïve and memory B-cells) and negatively with CD56 positive NK cells including different sub-populations (NK1 and NK2, for details see Auer *et al*.^3^). This underlines the suppression of anti-tumor NK cells in response to PD-L1 expression. All other analyzed immune cell populations^3^ were not significantly associated with PD-L1 expression (data not shown).

A similar analysis of systemic immune cell populations in blood revealed regulatory T-cells (Treg) and memory B-cells positively and HLA-DR positive NK4 cells negatively associated with PD-L1 abundances on tumor cells.

Analysis of immune cell populations^3^ in ascites revealed no significant associations with PD-L1 expression in EOC tumor tissue (data not shown).

### Correlation of PD-L1 expression with serum metabolites

A targeted and integrative serum metabolomics approach (Bachmayr-Heyda & Aust *et al*.^20^) with PD-L1 tumor cell expression in 19 primary and 17 metastatic tumor tissues of 22 patients as outcome revealed 65 out of 182 serum metabolites as significantly (FDR 5%) associated with PD-L1 abundance, thereof 56 glycerophosphilipids, four sphingolipids, three amino acids (his, trp, and tyr; the former two essential proteinogenic amino acids), and two biogenic amines (ADMA and putrescine). All–except the two latter biogenic amines–were positively correlated. Interestingly, these metabolites were highly overlapping (37 out of 65 significantly associated metabolites overlapped with the 54 significantly with OS associated metabolites from the OS metabolomics study^20^, p = 3.6 10^−9^, Suppl. Fig. S5) especially the glycerophospholipids (32 out of 65 overlapping with 43 from the OS metabolomics study) and amino acids (two out of three overlapping with four from the OS metabolomics study).

### PD-L1 in primary versus recurrent EOC

The use of TMAs to analyze PD-L1 expression in EOC has been previously published^17^ and allows a homogenous staining procedure. PD-L1 status shows a good concordance between small biopsies and resected specimens^21^; still, compared to WTSs only a small tumor area can be analyzed, this has to be considered as a limitation of the following analysis.

Among the different ovarian cancer histologies (Table 4), PD-L1 expression was observed in 21% (n = 8) and 15% (n = 5) in primary tissue (P) and metastatic implants (I), respectively, whereby percentage of PD-L1 positive tumor cells did not exceed 10%. In recurrence (RI), a total of 19 samples (39%) showed PD-L1 expression. Percentage of PD-L1 positive tumor cells reached up to 80% but only in one non-serous sample (RI). In the population of patients with a second recurrence (RII), 46% (n = 6) showed PD-L1 expression (with a maximum of 15% positive tumor cells).

### PD-L1 abundance increases in recurrence in serous EOC

**Figure 5:**
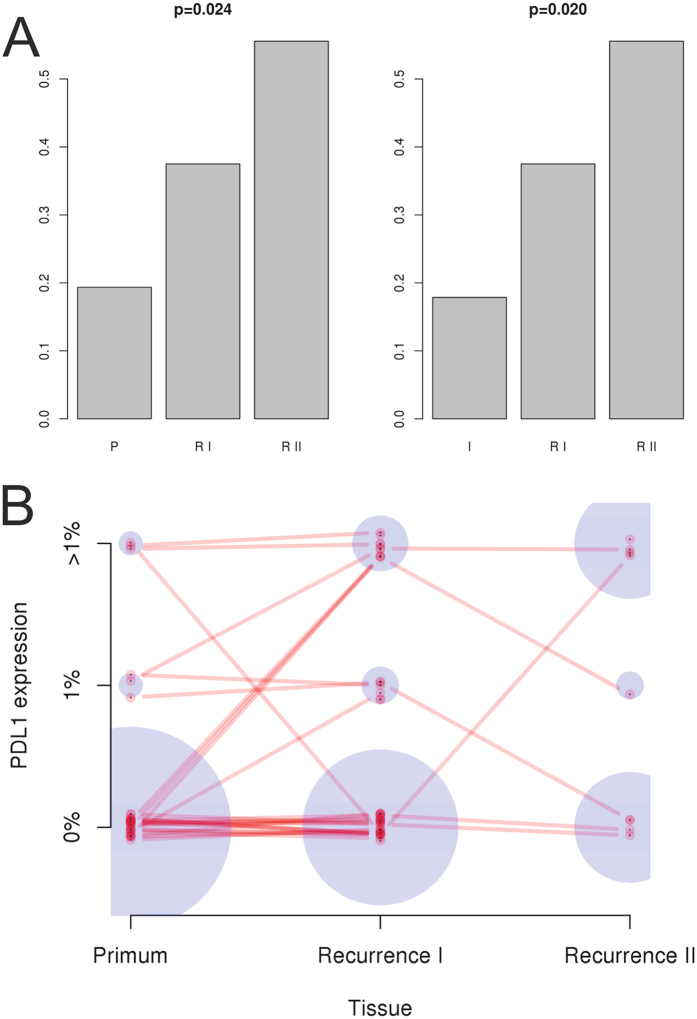
Course of PD-L1 expression in serous EOC comparing (**A**) primary tumor tissues of the ovary (P) or metastatic peritoneal implants (I) to tumor recurrences (first recurrence, RI or second recurrence, RII) (y-axis, ratio of positive cases) and (**B**) a scheme showing the course of PD-L1 abundances of all samples individually, connected if samples are from the same patient (blue circles represent relative frequencies at each position).

A steady and significant increase of PD-L1 abundance was seen in recurrence II (RII) compared to recurrence I (RI) and to primary tumors (P) in serous EOC as shown in Fig. 5A (p = 0.024). Out of the six samples positive in the primary tumor, five remained also positive in RI. As the origin of the recurrences is unknown, arising either from the ovarian tumor mass or the peritoneal implants, steady increasing PD-L1 abundance was also observed comparing RII to RI and to peritoneal implants (I, “metastasis”; p = 0.020; Fig. 5A). Taken together, in the subset of serous EOC, PD-L1 expression was seen in 19.4% (n = 6; maximum 10% positive tumor cells), 17.9% (n = 5; maximum 10% positive tumor cells), 37.5% (n = 15; maximum 30% positive tumor cells), and 55.6% (n = 5, with up to 15% positive tumor cells), in P, I, RI, and RII, respectively.

### PD-1 in primary versus recurrent serous EOC

Comparing PD-1 expression on TILs between P, RI, and RII serous EOC showed a slight but not significant increase (68.7% vs 71,1% vs 80.0%, respectively; p = 0.537) the same was seen comparing I to RI and RII (data not shown).

## Discussion

Our study revealed a significant positive association between PD-L1 abundances and the expressions of MHC I genes in tumor cells of high grade serous ovarian cancer. Therefore, we propose two different strategies of immune-evasion in HGSOC, either the upregulation of PD-L1 on tumor cells to inhibit T-cell cytotoxic activity or the down-regulation of MHC I aiming to avoid antigen presentation and thus T-cell recognition. The latter accompanied by a more malignant phenotype with worse overall survival. Additionally, this is the first study comparing PD-L1 expression in primary versus recurrent tumor tissues of serous EOC, showing a significantly increased expression of PD-L1 in tumor recurrences.

In our study, all expressed MHC I genes, *i.e.* HLA-A, HLA-B, HLA-C, HLA-E, HLA-F, HLA-G, HLA-DMA, and also B2M, were significantly positively correlated to PD-L1 expression. This data reflects the diversity in immune escape mechanisms in EOC in a selected population of only advanced stage HGSOC patients. Antigen presentation by human leukocyte antigen (HLA) molecules is required for detection and fighting of cancer cells by cytotoxic T-cells. Downregulation of HLA class I expression is a common immune-escape mechanism of tumor cells^22^. Tumors undergo a CD8+ T-cell mediated selection process and a down-regulation of MHC I expression in advanced stages^23^ – in EOC we see two parallel and mutually exclusive ways of immune escape: we hypothesize that EOC tumor cells are either (i) activating the immune regulatory mechanism of PD-L1 expression to inhibit T cell receptor signaling or (ii) down-regulate MHC I molecules as seen in PD-L1 negative tumor tissues so that the immune system lacks an initiating signal (tumor-associated antigens).

In MHC class I positive tumors a higher density of CD8+ T-cell infiltration was already described^24^ and correspondingly we also observed a higher number of CD8+ T-cells in PD-L1 positive tumors. Both, PD-L1 expression but also a high degree of tumor infiltrating cytotoxic cells are considered positive prognostic factors in EOC^7^,^16^. High CD8+ cell infiltration might trigger PD-L1 expression on tumor cells as an immune escape mechanism during cancer progression and evolution. As a result, PD-1 expressing CD8+ cells loose functional antitumor activity^16^ – therefore CD8 positive cells as potential prognostic biomarker need to be further characterized to truly understand their positive prognostic impact. We found PD-1 positive TILs in all samples with PD-L1 positive tumor cells. Absence of PD-1 positive TILs was only observed in PD-L1 negative tumor tissues.

PD-L1 abundance was not only positively associated with CD8+ T-cell but also with B-cell tumor-infiltration. In blood, also regulatory T-cells were positively associated with high PD-L1 tumor cell abundances, indicating a systemic down-regulation of T-cell mediated immunity. PD-L1 tumor abundance was negatively correlated with the serum cytokine IL10 and positively correlated with CCL22. IL10 suppresses proinflammatory cytokine secretion, antigen presentation and CD4+ T cell activation and CCL22 is associated with regulatory T-cell migration in ovarian cancer.

A negative association of PD-L1 abundance with (specific) NK cells was not only seen in in tumor tissues but also systemically in blood. Although NK cells seem to have limited effects in established solid tumors^25^, they are interesting targets of adoptive immunotherapy as they have the ability to interact with tumor cells that express very low levels of MHC I^26^,^27^. Thus, adoptive NK cell immunotherapy might be interesting for a selected population of patients with PD-L1 negative EOC tissues – probably less responsive for checkpoint inhibitors? Still, this approach has to be considered with caution as the only clinical trial on NK cell therapy revealed severe toxicities^28^.

In a previous study^20^ we showed that especially (poly)unsaturated glycerophospholipids in serum were associated positively with (i) favorable survival, (ii) corresponding concentrations in ascites and tumor tissues, (iii) respiratory chain gene expressions, and (iv) histones expressions and negatively (v) with systemic inflammation and vi) fatty acid neosynthesis. Most striking was the positive correlation with the expression of many of the histones genes, which were themselves significantly positively correlated to the number of expressed genes and transcriptional noise. We interpreted this “many-genes-expressed” phenotype as a putative neo-pluripotent state of the tumor cells, granting the tumor a highly malignant phenotype with unfavorable overall survival. Correlating these results to the PD-L1 and MHC I expression levels shown in this study reveals the following picture: Tumors with this neo-pluripotent malignant phenotype are MHC I low expressing tumors thus escaping the adaptive immune reaction by down-regulation of antigen presentation.

This data shows, in accordance with a previously published review^29^, that targeting immune-escape mechanisms is complex and various pathways have to be considered simultaneously. Comparing primary tumor tissue from the primary tumor (ovary) to tumor tissues acquired in the setting of tumor recurrence (or consecutive recurrences) revealed a significant increase of PD-L1 in relapsed serous EOC (p = 0.024). Since tumor tissues from relapsed EOC were taken from various sites we also compared them to metastatic implants within the peritoneal cavity, revealing a similar significant increase in PD-L1 expression (p = 0.020). Although this data was acquired in a small patient population and although PD-L1 expression on tumor cells seems to be not a reliable predictive marker of response to checkpoint inhibitors yet^15^, we could show that in EOC both, MHC I down-regulation and PD-L1 expression need to be considered concurrently.

## Conclusion

The immune-suppressive function of the PD-1/PD-L1 immune checkpoint pathway emerges as a promising oncologic target in a variety of cancer types. The heterogeneity between tumor entities as well as the heterogeneity seen within one tumor type consequently results in different response patterns. Inevitably, the clinical challenge remains again in overcoming temptation to draw premature conclusions as data obtained within different cancer types might not apply for all tumor entities. In EOC we still need to define criteria to guide patient selection for PD-L1 therapy.

## Material and Methods

### Study population and tumor tissues

To perform a deep analysis of PD-L1 expression and associated biological processes, a previously described and extensively analyzed prospectively collected cohort of 28 HGSOC patients with available gene expression data was used^2^,^3^,^4^. Ascites, fresh and FFPE tumor tissues, and blood were available for further analyses (summarized in Table 1). Paired whole tissue sections (WTS) from ovarian tumor tissue (P, for “primary”) and from metastatic implants within the peritoneal cavity (M, for “metastasis”) were employed for immunohistochemistry (IHC). Gene expression data obtained from matched isolated and EpCAM enriched tumor cells from fresh tumor tissues was correlated to PD-L1 abundances on corresponding FFPE slides.

To evaluate PD-L1 abundance in primary versus recurrent EOC, a second patient cohort was employed including only EOC patients undergoing surgery at tumor recurrence at the Medical University of Vienna between 1995 and 2015. In total, 64 patients with a median age at diagnosis of 55 years (range 24–82 yrs) were included in the final analyses (Table 4). Some patients underwent primary cytoreductive surgery in external hospitals. In these cases, only clinical data but no tissue samples from primary surgery were available. Retrospectively, FFPE tissues were collected from the time of tumor recurrence (first recurrence RI, n = 49; second recurrence RII, n = 13) as well as the time of primary surgery (P, n = 46). Samples from patients who received neoadjuvant chemotherapy prior to primary surgery were excluded. Paired samples with tumor tissues from primary tumor and recurrence were available from 31 patients and thereof matched samples from a second recurrence were available in seven patients. Comparison of PD-L1, PD-1, and CD8 expression was performed using tissue microarrays (TMAs), constructed according to standard procedures comprised of three one millimeter cores per tumor tissue. Approval for this study was obtained by the ethical review board of the Medical University of Vienna (nos 366/2003 and 793/2011) and all patients signed an informed consent. All experiments were conducted according to the “Good Scientific Practice” guidelines from the Medical University of Vienna, Version 2, 2012.

### Immunohistochemistry

Staining was performed using a validated anti-PD-L1 antibody (PD-L1 clone E1L3N XP^®^ Rabbit mAb, Cell Signaling Technology, Danvers, MA, USA). This antibody had already been used successfully with EOC tissues^16^, showing more reliable results compared to previously described anti-PD-L1 antibodies^17^,^30^. To test the accuracy of this specific PD-L1 antibody, a second antibody (VENTANA PD-L1 (SP263) Rabbit Monoclonal Primary Antibody) was used on two different test slides showing a highly similar staining pattern. Additionally, staining for PD-1 (Fig. 1B) and CD8 (Fig. 1C) was performed using the monoclonal mouse anti-PD-1 antibody (MRQ-22) from Cell Marque and the anti-CD8 monoclonal mouse antibody (Clone C8/144B) from Dako^7^. IHC was performed in a standardized setting using the Leica bond Polymer Refine Detection kit DS9800 according to staining procedures established at the Department of Pathology, Medical University of Vienna. PD-L1 expression of EOC tumor cells and tumor infiltrating lymphocytes (TILs) was evaluated. Percentage of the lymphocyte subset of CD8+ TILs and percentage of PD-1 positive lymphocytes of total CD8+ cells were analyzed. All quantitative assessments were done by two trained IHC analysts and discordant estimates resolved together.

### Luminex analyses

Cyto- and Chemokine analysis was performed using Luminex technology on a Bio-Plex 200 System (Bio-Rad Laboratories, Hercules, California, USA) in serum and cell-free ascites of the 28 HGSOC patients using a set (set one) of two Multiplexed Luminex-based assay panels (Bio-Plex Pro Human Cancer Biomarker Assays: Panel 1, and Bio-Plex Pro Human Chemokine Panel Assay; Bio-Rad Laboratories), and (set two) the Cytokine Human Magnetic 25-Plex Panel; Life Technologies. Luminex analyses were performed as previously described^3^,^31^. Cyto- and chemokine concentrations in serum and ascites were compared to logarithmized PD-L1 abundances in primary and metastatic tumor tissues using Bioconductor’s R-package limma v3.28.17 with its *lmFit* and *eBayes (trend* = *TRUE*) functions and tissue origin (P or M) as correcting factor^32^. A false discovery rate (FDR) cut-off of 20% was used.

### Flow cytometric analysis

Immune cell compositions of digested and tumor cell depleted fresh tumor tissues, serum, and cell-free ascites were estimated using two six-color flow cytometric staining panels on a BD FACSVerse flow cytometer, equipped with three lasers (405 nm, 488 nm, and 633 nm) as described in Auer *et al*.^3^. Analysis was performed using FlowJo software (v7.6.2, Tree Star, Inc., Ashland, OR, USA). 26 different cell populations were quantified, including: CD3+ lymphocytes, separated (gated) into cytotoxic T-cells (CD8+), T-helper cells (Th, CD4+), regulatory T-cells (Tregs) and natural killer T-cells (NKT, HLA-DR+/−). CD3- lymphocytes were gated into B-cells (CD19+, naïve and memory), natural killer (NK) cells (CD56+), and five different subsets of NK cells (each HLA-DR+/−. Cell numbers are given as relative frequencies and absolute values (if possible; named “a …”). For further details see Auer *et al*.^3^. In total, 43 relative and absolute immune cell frequencies were compared to logarithmized PD-L1 abundances as described in the Luminex analyses section.

### Metabolomics

Targeted metabolomics in serum was performed using AbsoluteIDQ p180 kits (Biocrates Life Sciences AG, Innsbruck, Austria)^20^. The kit allows the identification and (semi-) quantification of metabolites by liquid chromatography (LC)- and flow injection analysis (FIA)-multiple reaction monitoring (MRM). The samples were analyzed on an AB SCIEX QTrap 4000 mass spectrometer (Framingham, MA, USA) using an Agilent 1200 RR HPLC system (Agilent Technologies, Santa Clara, CA, USA), which were operated with Analyst 1.6.2 (AB SCIEX). The chromatographic column was obtained from Biocrates. The serum samples and additional blanks, calibration standards and quality controls were prepared according to the user manual. All amino acids and biogenic amines were derivatized with phenylisothiocyanate. The experiments were validated using the supplied software (MetIDQ, Version 5-4-8-DB100-Boron-2607, Biocrates). Metabolite concentrations were compared to PD-L1 abundances as described in the Luminex analyses section. A false discovery rate (FDR) cut-off of 5% was used.

### RNA sequencing

rRNA depleted total RNA from 17 tumor cell enriched tissue samples (ovarian and peritoneal masses) from 15 patients were sequenced to a median depth of 26.03 million 50 bp paired-end reads (range: 10.67–44.65 million). Fresh tumor tissue was enzymatically digested and enriched for EpCAM positivity to receive a single-tumor cell-suspension with only minimal contamination by immune cells and stromal cells. Sequencing details are already described in Auer *et al*.^2^. Raw read counts were cyclic-loess normalized and genes’ and samples’ weights calculated by the voomWithQualityWeights function from the limma Biconductor-package^33^. Comparing gene expression from solid tumors from the ovary (P, n = 9) and implants from the peritoneum (M, n = 8) revealed no significant difference, therefore P and M samples were analyzed together but in the design model accounted for. The dependent variable was the logarithmized PD-L1 abundance. A false discovery rate (FDR) cut-off of 20% was used.

## Additional Information

**How to cite this article:** Aust, S. *et al*. Absence of PD-L1 on tumor cells is associated with reduced MHC I expression and PD-L1 expression increases in recurrent serous ovarian cancer. *Sci. Rep.* **7**, 42929; doi: 10.1038/srep42929 (2017).

**Publisher's note:** Springer Nature remains neutral with regard to jurisdictional claims in published maps and institutional affiliations.

## Supplementary Material

Supplementary Figures

## Figures and Tables

**Table 1:** Characteristics of the HGSOC sample (n = 28); PD-L1 (%) indicates PL-L1 positive tumor cell abundance in %.

ID	Age (yrs)	FIGO	Grade	LN	Ascites	PD-L1 (%)
3	54	IIIC	G3		>500	5
5	80	IIIC	G2	1	<500	10
6	70	IIIC	G2		>500	0
9	62	IIIC	G2		>500	5
12	55	IIIC	G3	1	>500	0
13	60	IIIC	G3	1	>500	0
16	64	IIIC	G3		>500	0
24	50	IIIB	G2		>500	5
25	50	IIIC	G3	1	>500	0
27	66	IIIA	G3	0	0	5
28	57	IIIC	G3		<500	1
29	53	IIIC	G3	0	<500	5
30	41	IIIC	G3	0	<500	5
31	69	IIIB	G3		0	5
35	56	IIIB	G3		0	5
39	50	IIIC	G3	0	0	5
41	49	IIC	G3	0	>500	5
42	49	IIIB	G3	0	>500	5
53	48	IIIC	G3	1	0	20
54	34	IV	G3		>500	0
55	81	IIA	G3	0	<500	0
58	66	IIIC	G3	0	0	1
60	50	IIIC	G3	0	>500	5
62	54	IIIC	G3	1	<500	5
65	44	IIIC	G3	1	>500	5
81	60	IV	G3	1	<500	5
84	52	IV	G3	1	>500	0
85	46	IIIC	G3		<500	0

LN, malignant lymph nodes.

**Table 2:** Pathways significantly associated with PD-L1 expression in HGSOC tumor cells (FDR 20%).

Name	ID^19^	pSize	NDE	FDR	Status
Antigen processing and presentation	4612	57	6	0.1	Activated
Cytokine-cytokine receptor interaction	4060	192	8	0.1	Activated
Chemokine signaling pathway	4062	157	5	7.3	Activated

*Cf.* Fig. 2A.

**Table 3:** Cytokines and chemokines significantly associated with PD-L1 expression: serum HER-2/neu (sHER2neu), C-C chemokine ligand 22 (CCL22), soluble epidermal growth factor receptor (sEGFR), Granulocyte-macrophage colony-stimulating factor (GM-CSF), C-C chemokine ligand 23 (CCL23), platelet endothelial cell adhesion molecule (PECAM-1), C-X-C motif chemokine 11 (CXCL11), macrophage migration inhibitory factor (MIF), interleukin 5 (IL5), interleukin 2 receptor (IL2R), interalukin 10 (IL10).

ID	logFC	Abundance	FDR
Set one			
sHER2neu	0.13	13.11	8.9
CCL22	0.17	9.56	9.6
Leptin	0.52	12.45	9.6
sEGFR	0.13	14.77	9.6
GM-CSF	0.27	5.83	9.6
CCL23	−0.19	8.50	9.6
PECAM-1	0.07	11.80	9.6
CXCL11	−0.24	5.78	14.8
MIF	0.45	11.17	17.8
Set two			
IL5	−0.28	1.72	12.7
IL2R	−0.32	8.27	12.8
IL10	−0.36	2.84	12.8

LogFC, log_2_ fold change correlating with log_2_ PD-L1 abundance values; Abundance, mean log_2_ abundance in serum; FDR, false discovery rate (%).

**Table 4:** Clinical characteristics of patients receiving surgery at tumor recurrence.

Primary tumor (n = 46)			
Characteristics	n (%)	Characteristics	n (%)
Histology		Grade	
Serous	37 (80)	Grade 1	11 (24)
Non-serous	9 (20)	Grade 2	12 (26)
Residual tumor		Grade 3	23 (50)
no	39 (85)	FIGO	
>1 cm	7 (15)	I	2 (4)
Response*		II	7 (15)
CR	40 (87)	III	32 (70)
PR	5 (13)	IV	5 (11)
**Recurrence I (n = 49)**		**Recurrence II (n = 13)**	
Histology		Histology	
Serous	40 (82)	Serous	9 (89)
Non-serous	9 (18)	Non-serous	4 (31)

In some cases surgery of primary or first tumor recurrence was executed in external hospitals, therefore tissue samples were not available. Only data of patients included in the TMA analyses are shown.

^*^CR, complete response; PR, partial response; one missing.
