# Supplementary material for: Absence of PD-L1 on tumor cells is associated with reduced MHC I expression and PD-L1 expression increases in recurrent serous ovarian cancer
Source: Sci Rep. 2017 Mar 7;7:42929. doi: 10.1038/srep42929 (PMC5339797; doi:10.1038/srep42929)
Supplement: Supplementary Figures [file srep42929-s1.pdf]

Absence of PD-L1 on tumor cells is associated with reduced MHC I expression and PD-L1 expression increases in recurrent serous ovarian cancer

– Supplementary Figures –

Stefanie Aust[1], Sophie Felix[1], Katharina Auer[1], Anna Bachmayr-Heyda[1], Lukas Kenner[2], Sabine Dekan[2], Samuel M. Meier[3], Christopher Gerner[3], Christoph Grimm[1], Dietmar Pils[4,5,*]

[1]Dept. of Obstetrics and Gynecology, Comprehensive Cancer Center (CCC), Medical University of Vienna, Vienna, Austria
[2]Dept. of Pathology, Medical University of Vienna, Vienna, Austria
[3]Dept. of Analytical Chemistry, University of Vienna, Vienna, Austria
[4]Center for Medical Statistics, Informatics, and Intelligent Systems (CeMSIIS), Medical University of Vienna, Vienna, Austria
[5]Dept. of Surgery, Medical University of Vienna, Vienna, Austria

*Email: dietmar.pils@univie.ac.at

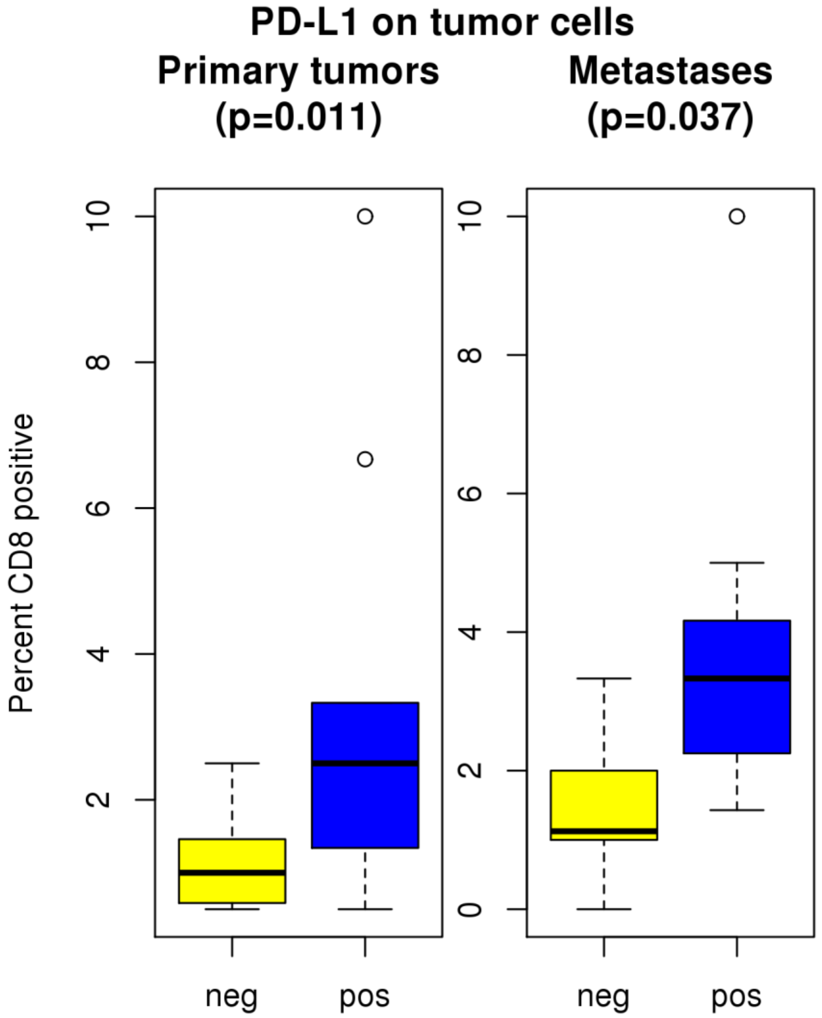

Supplementary Figure S1. Percentages of CD8 positive TILs in PD-L1 negative (neg) and positive (pos) primary and metastatic tumor tissues (P-values, T-Tests).

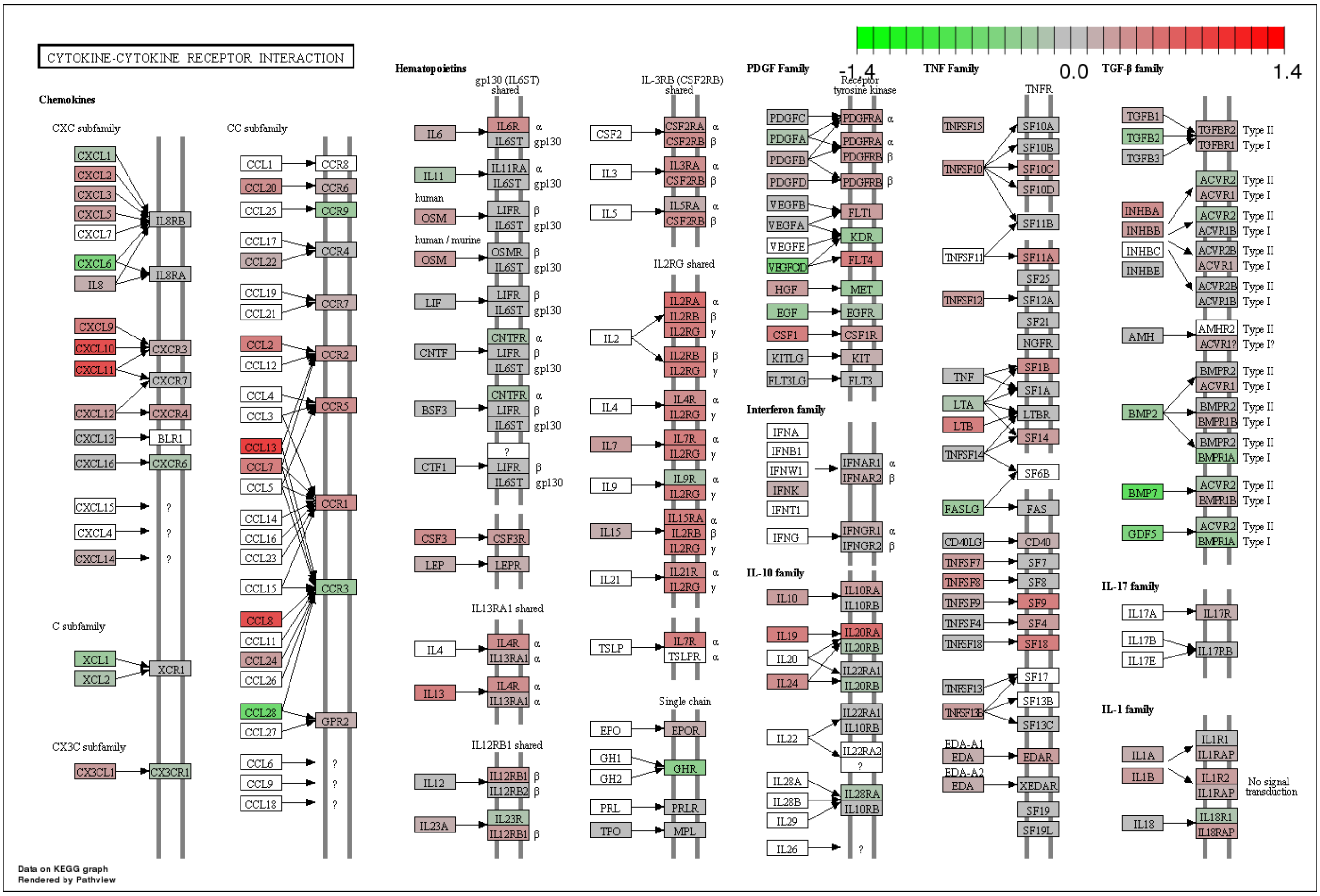

CYTOKINE-CYTOKINE RECEPTOR INTERACTION
-1.4
0.0
1.4
Chemokines
CXC subfamily
CXCL1
CXCL2
CXCL3
CXCL5
CXCL7
CXCL6
IL8
IL8RB
IL8RA
CXCL9
CXCL10
CXCL11
CXCR3
CXCR7
CXCL12
CXCR4
CXCL13
BLR1
CXCL16
CXCR6
CXCL15
CXCL4
CXCL14
?
CC subfamily
CCL1
CCR8
CCL20
CCR6
CCL25
CCR9
CCL17
CCL22
CCR4
CCL19
CCL21
CCR7
CCL2
CCL12
CCR2
CCL4
CCL3
CCR5
CCL13
CCL7
CCL5
CCL14
CCL16
CCL23
CCR1
CCL15
CCR3
CCL8
CCL11
CCL24
CCL26
CCL28
CCL27
GPR2
CCL6
CCL9
CCL18
C subfamily
XCL1
XCL2
XCR1
CX3C subfamily
CX3CL1
CX3CR1
Hematopoietins
gp130 (IL6ST) shared
IL6
IL6R
IL6ST
gp130
IL11
IL11RA
human
OSM
LIFR
human / murine
OSMR
LIF
CNTF
CNTFR
BSF3
CTF1
CSF3
CSF3R
LEP
LEPR
IL13RA1 shared
IL4
IL4R
IL13RA1
IL13
IL12RB1 shared
IL12
IL12RB1
IL12RB2
IL23A
IL23R
IL-3RB (CSF2RB) shared
CSF2
CSF2RA
CSF2RB
IL3
IL3RA
IL5
IL5RA
IL2RG shared
IL2
IL2RA
IL2RB
IL2RG
IL4R
IL7
IL7R
IL9
IL9R
IL15
IL15RA
IL21
IL21R
TSLP
TSLPR
Single chain
EPO
EPOR
GH1
GH2
GHR
PRL
PRLR
TPO
MPL
PDGF Family
Receptor tyrosine kinase
PDGFC
PDGFRA
PDGFA
PDGFB
PDGFRB
PDGFD
VEGFB
FLT1
VEGFA
KDR
VEGFE
VEGFD
FLT4
HGF
MET
EGF
EGFR
CSF1
CSF1R
KITLG
KIT
FLT3LG
FLT3
Interferon family
IFNA
IFNB1
IFNW1
IFNK
IFNT1
IFNAR1
IFNAR2
IFNG
IFNGR1
IFNGR2
IL-10 family
IL10
IL10RA
IL10RB
IL19
IL20
IL24
IL20RA
IL20RB
IL22RA1
IL22
IL22RA2
IL28A
IL28B
IL29
IL28RA
IL26
TNF Family
TNFR
TNFSF15
TNFSF10
TNFSF11
TNFSF12
TNF
LTA
LTB
TNFSF14
FASLG
CD40LG
TNFSF7
TNFSF8
TNFSF9
TNFSF4
TNFSF18
TNFSF13
TNFSF13B
EDA-A1
EDA
EDA-A2
SF10A
SF10B
SF10C
SF10D
SF11B
SF11A
SF25
SF12A
SF21
NGFR
SF1B
SF1A
LTBR
SF14
SF6B
FAS
CD40
SF7
SF8
SF9
SF4
SF18
SF17
SF13B
SF13C
EDAR
XEDAR
SF19
SF19L
TGF-β family
TGFB1
TGFB2
TGFB3
TGFBR2
TGFBR1
Type II
Type I
INHBA
INHBB
INHBC
INHBE
ACVR2
ACVR1
ACVR2B
ACVR1B
AMH
AMHR2
ACVR1?
Type I?
BMP2
BMPR2
BMPR1B
BMPR1A
BMP7
GDF5
IL-17 family
IL17A
IL17R
IL17B
IL17E
IL17RB
IL-1 family
IL1A
IL1R1
IL1RAP
IL1B
IL1R2
No signal transduction
IL18
IL18R1
IL18RAP
Data on KEGG graph
Rendered by Pathview

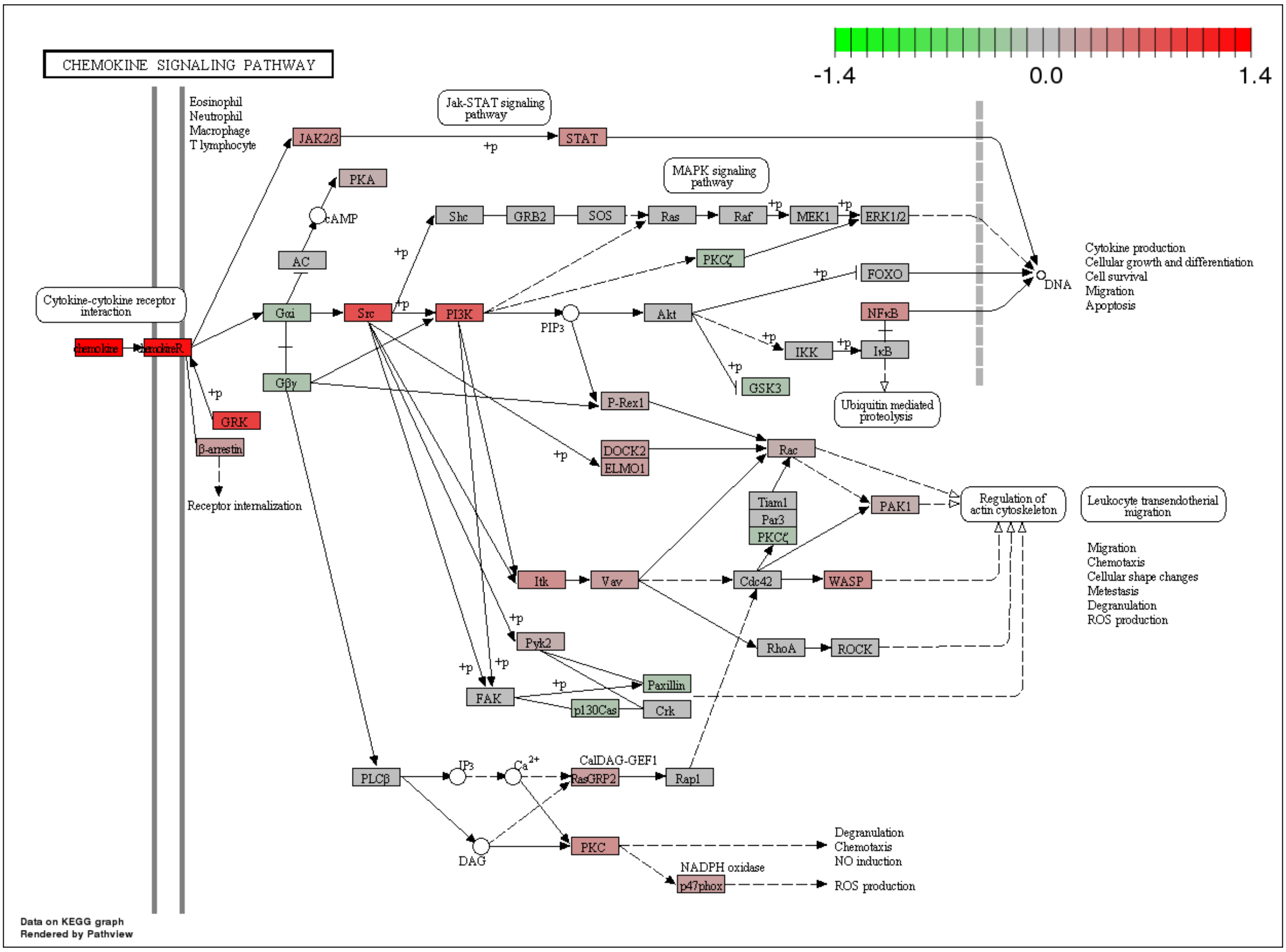

Supplementary Figure S2. Significantly deregulated pathways in tumor cells, associated with PD-L1 abundance on tumor cells. (http://www.kegg.jp/kegg/kegg1.html[1])

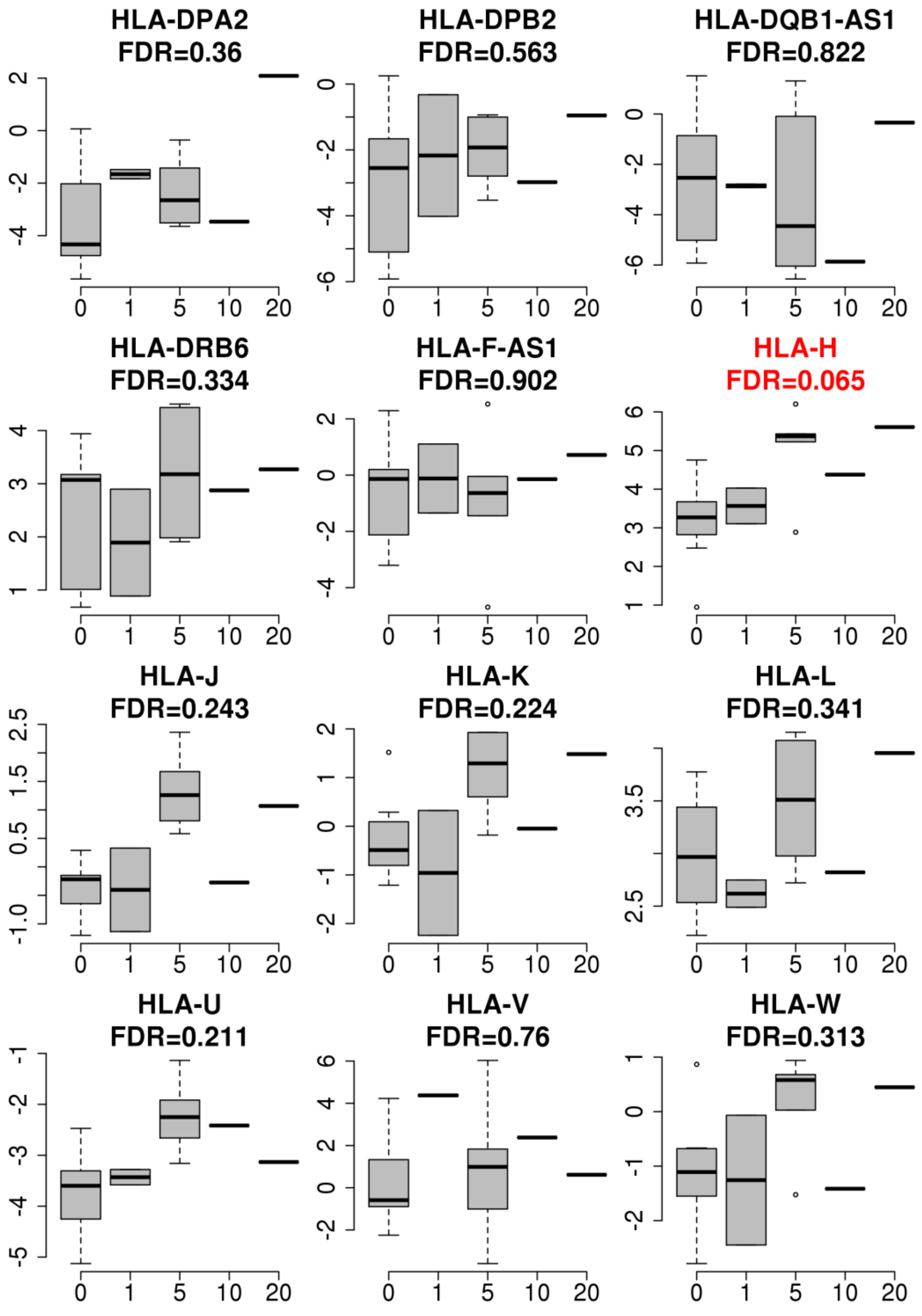

Supplementary Figure S3. Correlation of non-coding and antisense (-AS1) HLA genes with PD-L1 abundance on tumor cells. X-axis, percentage of PD-L1 positive tumor cells; y-axis, relative log_2 expression; red, significant association (FDR <20%).

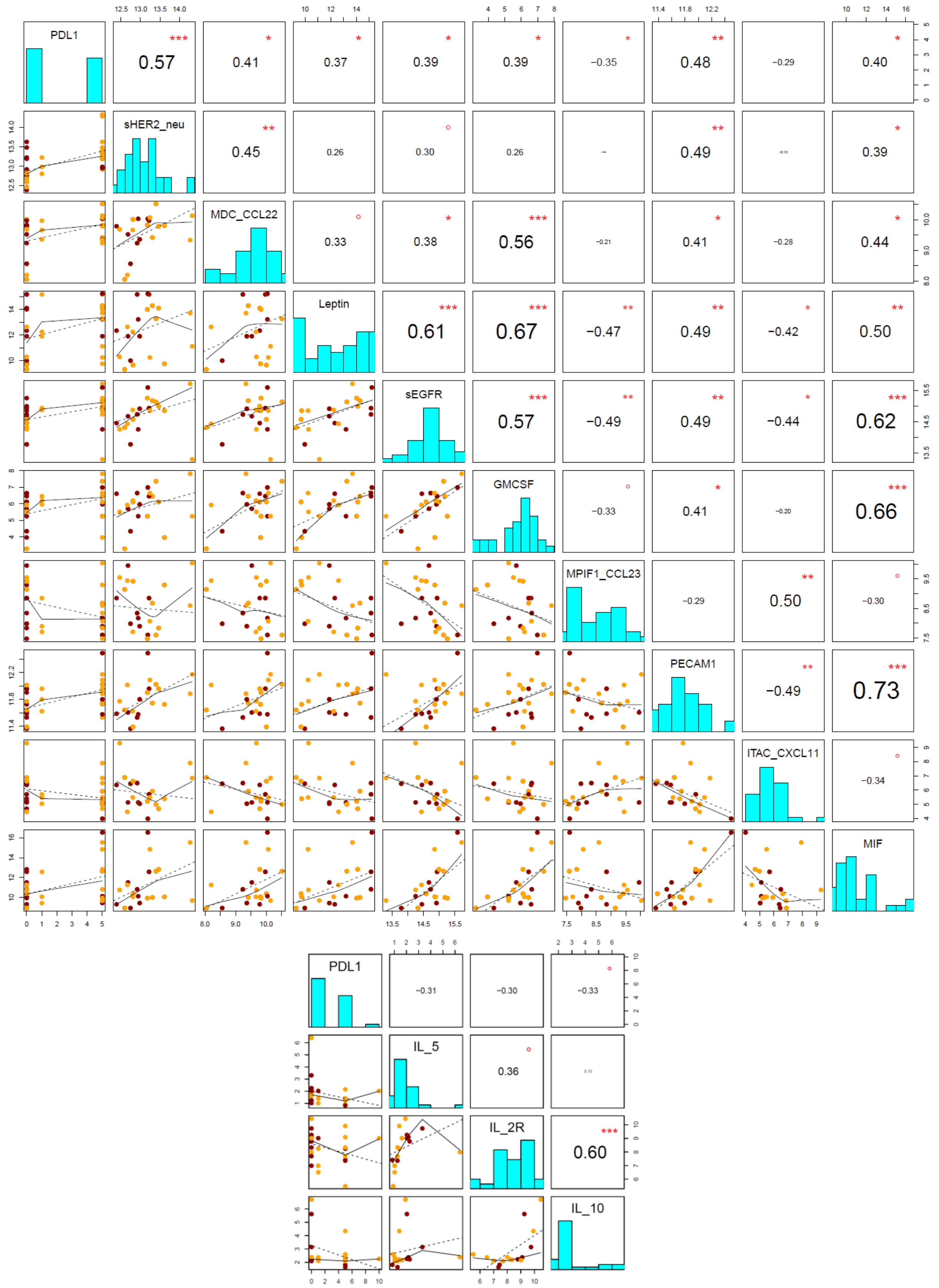

Supplementary Figure S4. Serum cyto- and chemokines significantly associated with PD-L1 abundance on tumor cells. Correlation plots, R-values (given in top-right boxes) and significance levels (p < °0.1, *0.05, **0.01, ***0.001) are only illustrations. Statistical significance was calculated using multiple linear regressions with logarithmized PD-L1 abundance as dependent variable, corrected for tumor tissue; primary (red) or metastatic (orange). False Discovery Rates (FDRs) are given in Table 4.

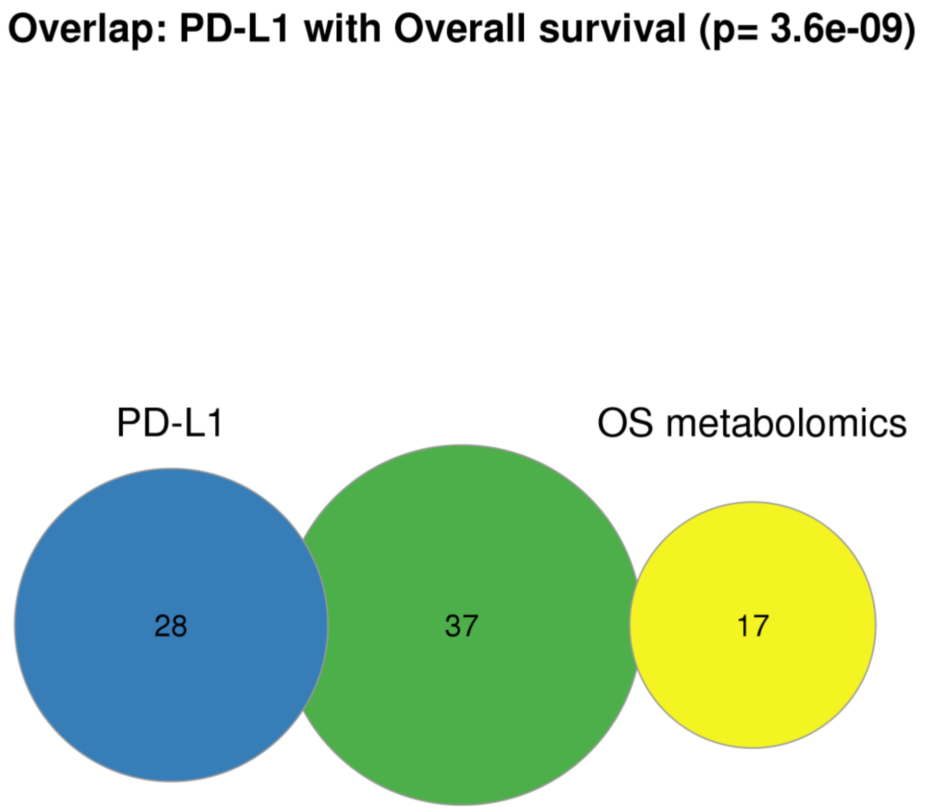

Supplementary Figure S5. Overlap of metabolites significantly positively associated with PD-L1 tumor cell expression ("PD-L1") and significantly negatively associated with unfavorable overall survival ("OS metabolomics" [2]) in HGSOC. P-value, hypergeometric overrepresentation test.

1 Kanehisa, M., Sato, Y., Kawashima, M., Furumichi, M. & Tanabe, M. KEGG as a reference resource for gene and protein annotation. *Nucleic Acids Res* **44**, D457-462, doi:10.1093/nar/gkv1070 (2016).

2 Bachmayr-Heyda, A. *et al.* Integrative Systemic and Local Metabolomics with Impact on Survival in High Grade Serous Ovarian Cancer. *Clin Cancer Res, DOI: 10.1158/1078-0432.CCR-16-1647 Published 19 October 2016* (2016).
